# Targeting matrix metallopeptidase 2 by hydroxyurea selectively kills acute myeloid mixed-lineage leukemia

**DOI:** 10.1038/s41420-022-00989-4

**Published:** 2022-04-08

**Authors:** Ruiheng Wang, Shufeng Xie, Shouhai Zhu, Yong Sun, Bowen Shi, Dan Li, Ting Kang, Yuanli Wang, Zhenshu Xu, Han Liu

**Affiliations:** 1 Shanghai Institute of Hematology, State Key laboratory of Medical Genomics, National Research Center for Translational Medicine at Shanghai, Ruijin Hospital, Shanghai Jiao Tong University School of Medicine, Shanghai, China; 2 Department of Radiology, Ruijin Hospital, Shanghai Jiao Tong University School of Medicine, Shanghai, China; 3 Department of Oncology, Xin Hua Hospital, School of Medicine, Shanghai Jiao Tong University, Shanghai, 200092 China; 4 Fujian Institute of Hematology, Fujian Provincial Key Laboratory of Hematology, Fujian Medical University Union Hospital, 350001 Fuzhou, China

**Keywords:** Acute myeloid leukaemia, Cell death

## Abstract

Oncogene-induced tumorigenesis results in the variation of epigenetic modifications, and in addition to promoting cell immortalization, cancer cells undergo more intense cellular stress than normal cells and depend on other support genes for survival. Chromosomal translocations of mixed-lineage leukemia (MLL) induce aggressive leukemias with an inferior prognosis. Unfortunately, most MLL-rearranged (MLL-r) leukemias are resistant to conventional chemotherapies. Here, we showed that hydroxyurea (HU) could kill MLL-r acute myeloid leukemia (AML) cells through the necroptosis process. HU target these cells by matrix metallopeptidase 2 (MMP2) deficiency rather than subordinate ribonucleotide reductase regulatory subunit M2 (RRM2) inhibition, where MLL directly regulates MMP2 expression and is decreased in most MLL-r AMLs. Moreover, iron chelation of HU is also indispensable for inducing cell stress, and MMP2 is the support factor to protect cells from death. Our preliminary study indicates that MMP2 might play a role in the nonsense-mediated mRNA decay pathway that prevents activation of unfolding protein response under innocuous endoplasmic reticulum stress. Hence, these results reveal a possible strategy of HU application in MLL-r AML treatment and shed new light upon HU repurposing.

## Introduction

Neoplastic transformation of human blood cells during hematopoiesis results from intricate genetic and epigenetic alterations [1]. Oncogenes precisely control malignant hematopoietic progenitors proliferation and block their lineage differentiation, practically by dysregulating genes related to anti-apoptosis, survival signals, cell cycle [2], and stemness maintenance [3]. Continuous activated oncogenic signaling is indispensable for the survival of certain tumor cell types, and directly targeting the cancer driver effectively leads to the collapse of tumor cell survival dependency on the oncogene [4]. Most mutations, especially loss-of-function mutations, currently found in tumor cells are not directly druggable [5]. Nevertheless, tumorigenesis usually left elevated stress on cancer cells, which instigated their survival dependency on other supporting factors [4, 6]. Targeting the genes assisting in stress relief is a practical approach to develop anti-cancer drugs.

Leukemia caused by chromosome translocations of mixed-lineage leukemia (MLL) is characterized with poor prognosis. MLL rearrangements occur in 5–10% of acute leukemia, primarily in infant and therapy-related secondary leukemia [7, 8]. More than 70 MLL partners have been identified, and the predominant genes associated with fusion are *AF4*, *AF9*, and *ENL*, accounting for 69% of this type of leukemia [9]. *MLL-AF4* prevalently induces pro-B acute lymphoblastic leukemia in humans, whereas *MLL-AF9* leads to acute myloid leukemia (AML) [10]. We previously targeted the proteotoxic stress of MLL-rearranged (MLL-r) leukemia with bortezomib, and the proteasome inhibitor displayed selective killing in acute lymphoblastic leukemia but nonspecific toxicity to AML cells, revealing that oncogene might exert different stress on distinct cell types [11]. Hence, efforts were spent on exploring novel chemotherapies due to the lack of adequate drugs for MLL-r AML, of which most current agents were devised to target the MLL complex.

DOT1L is a histone methyltransferase enzyme for trimethylation of H3K79, and the core component of the DOT1L complex [12]. MLL fusion up-regulates leukemic drive genes more requires H3K79 methylation at the MLL targets than non-MLL leukemia. Treatment with DOT1L inhibitors could likely kill MLL-r leukemia cell lines and displayed less toxicity to germline MLL counterparts [13]. Menin, a critical co-factor of MLL, interacts with MLL N-terminal to recruit the complex at the *HOXA9* locus. Studies have demonstrated that abolishing menin and MLL interplay is promising for therapy of MLL-r leukemia [12, 14]. H3K4 trimethylation is essential for MLL fusion mediated leukemogenesis. Disrupting the interaction of WDR5 and wild-type (WT) MLL, expressed by non-translocated allele, would inhibit MLL methyltransferase activity and induce apoptosis of MLL-r leukemia cells [15]. Recently, a significant study showed that targeting the IRAK4 pathway could prevent the degradation of WT MLL and promoted differentiation of MLL-r leukemia cells [16].

Our earlier finding showed that MLL played an important role in checkpoint of late DNA replication origin firing via H3K4 trimethylation [17]. We considered that MLL-r AML cells were haploid-insufficient to deal with excess DNA replication stress and vulnerable to the DNA replication inhibitor hydroxyurea (HU). The presented study elucidated the specific cell killing of HU in MLL-r AML cell lines through an alternative mechanism, targeting matrix metallopeptidase 2 (MMP2), where inhibition of ribonucleotide reductase regulatory subunit M2 (RRM2) was not the primary factor. These findings provided a new sight in this drug.

## Results

### MLL-AF9 AML cells are sensitive to HU treatment

**Fig. 1:**
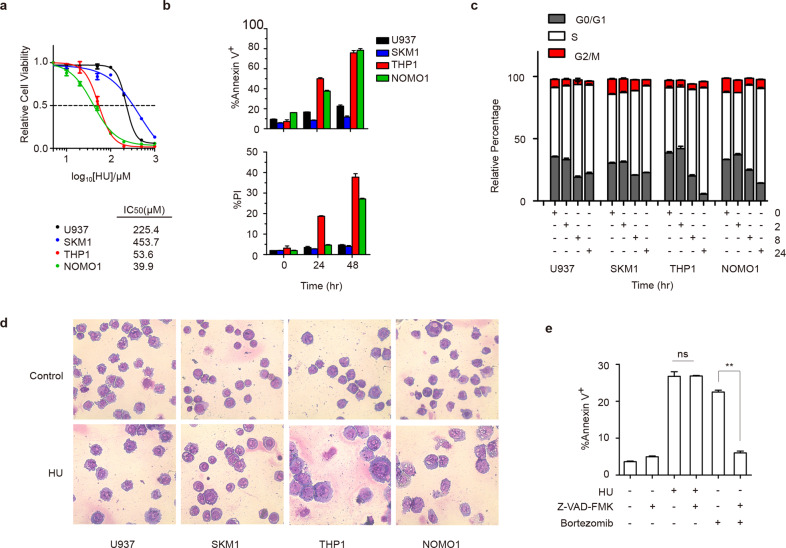
MLL-AF9 AML cells are sensitive to HU treatment. **a** Cell viability of indicated AML cell lines with HU treatment after 72 h. The IC_50_ of different cells was quantified. **b** Cells with 100 μM HU treatment were stained using Annexin V and propidium iodide. **c** Cell cycle profiles were analyzed upon indicated hours of exposure to 100 μM HU. **d** Cells were stained with Wright-Giemsa staining upon 24 h treatment with 100 μM HU. Magnification is 200×. **e** THP1 cells were pretreated with the apoptosis inhibitor 100 μM Z-VAD-FMK for 1 h, then treated with 100 μM HU or 10 nM bortezomib for 24 h, the viability was analyzed. **P < 0.01; the ns indicate no significant difference; two-tailed *t-*test. Data represent the means of triplicate reactions ± SD.

To verify whether MLL-r AML cells are sensitive to DNA replication inhibitors, HU was used to treat MLL germline, U937 and SKM1, and MLL-AF9, THP1 and NOMO1, cell lines for 72 h. As a result, THP1 and NOMO1 cells were susceptible to HU treatment (Fig. 1a). The MLL-AF9 cells showed higher levels of death than non-MLL leukemia cells with 100 μM HU treatment (Fig. 1b). HU induced cell cycle arrest partly by perturbing DNA replication and generating DNA damage in the S phase [17, 18]. We monitored the cell cycle change during early HU treatment and found all the cells displaying a reduction in G1 and G2/M phase, and a more excellent ratio of S phase (Fig.1c). Notably, the G1 phase of MLL-AF9 leukemia cells, furtherly diminished after 8 h, in contrast to those maintained in non-MLL leukemia cells. The BrdU incorporation heralded a very backward synthesis rate in MLL-AF9 leukemia cells upon the 2-hour treatment, while there was a recovery trend over time (Supplementary Fig. S1a).

We also sought to find whether cell differentiation occurred during HU treatment. Indeed, HU did not result in morphological differentiation into monocytic and granulocytic lineages of THP1 and NOMO1 cells, instead of causing tumescent nuclei and vacuole formation (Fig. 1d), which suggests they might suffer from necroptosis rather than apoptosis [19, 20]. Using the pan-caspase inhibitor Z-VAD-FMK could not prevent cell death under HU treatment (Fig. 1e). Loss of the mitochondrial membrane potential was also observed (Supplementary Fig. S1b). Altogether, these data demonstrated that HU is an effective drug targeting MLL-AF9 AML cells in vitro.

### RRM2 inhibition of HU is not principally responsible for MLL-AF9 cell killing

Most studies adopted HU to induce DNA damage for repair research [21–23]. Ribonucleotide Reductase is the only enzyme in eukaryote organisms responsible for controlling the source of dNTPs [24]. The complex consists of a catalytic RRM1 homodimer and a regulatory heterodimer of RRM2 and RRM2B, in which RRM2 is rate-limiting for enzyme activity during the S phase [25]. HU inactivates RRM2 by scavenging the iron-tyrosyl free radical, leading to a low or unbalanced dNTPs pool, further promoting replication stress and genomic instability [26].

**Fig. 2:**
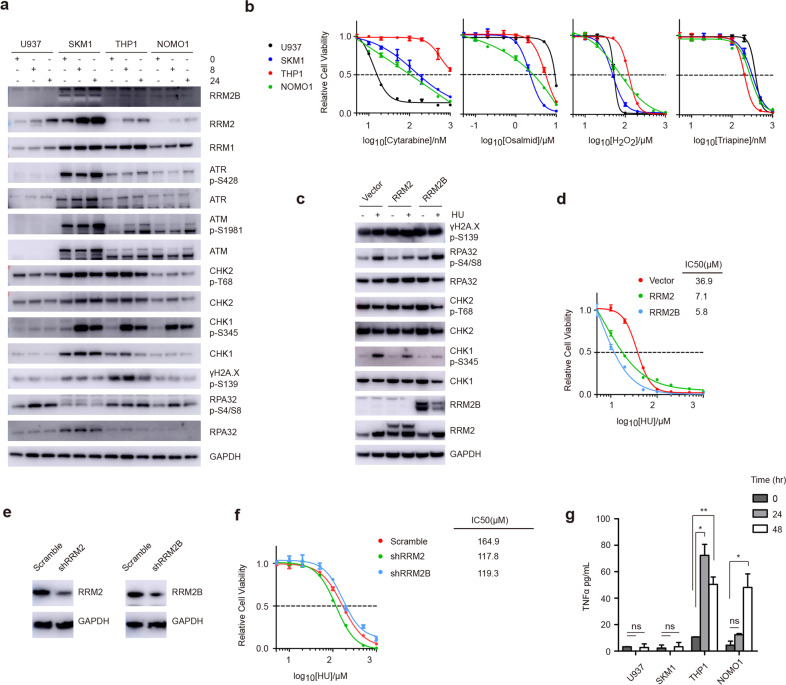
RRM2 inhibition by HU is not responsible for cell killing of AML cells harbouring MLL-AF9 proteins. **a** Immunoblots of indicated proteins in the AML cell lines at 0, 8, 24 h with 100 μM HU treatment. **b** Cell viability of treatment with indicated DNA damage agents after 72 h. **c**, **d** THP1 cells were infected with the indicated lentiviral vectors. Immunoblots (**c**) and cell viability (**d**) of the indicated cells were analyzed. The IC_50_ of different cells was quantified. **e**, **f** Knockdown of RRM2 or RRM2B was performed in U937 cells. Immunoblots (**e**) and cell viability (**f**) of the indicated cells were analyzed. The IC_50_ of different cells was quantified. **g** Concentration of TNFα in the supernatant of cells with 100 μM HU treatment after the indicated time. *P < 0.05; **P < 0.01; the ns indicate no significant difference; two-tailed t-test. Data represent the means of triplicate reactions ± SD.

We surmised MLL-AF9 cells defected in response to RRM2 inhibition and inspect the activation of DNA checkpoints and RRM2 level. The results show that CHK1 was strongly stimulated, accompanied by a progressive increase of RRM2 (Fig. 2a). This positive feedback loop regulation could be illustrated by that CHK1 raised *RRM2* expression in an E2F1-dependent manner [27]. Moreover, although RRM2 was highly expressed in SKM1, no obvious difference was observed between U937 and THP1 cells. Significantly, the γH2A.X and phosphorylated-RPA32 levels were stable, implying that no DNA damage accumulated during HU treatment (Fig. 2a).

Several other DNA damage agents were used to treat these cell lines. Osalmid is a novel RRM2 inhibitor by binding to the hydrogen bond of RRM2 [28], and triapine is an iron chelator, similar to the action of HU [29]. Unexpectedly, all agents failed to recur the cell survival pattern of HU (Fig. 2b), manifesting that DNA toxicity and RRM2 inhibition were not the primary factors accounting for the susceptibility of MLL-AF9 cells to HU treatment. Besides, a high concentration of HU caused a non-replicating S phase in THP1 (Supplementary Fig. S2a), indicating the inability of 100 μM dosage to dysfunctional RRM2. Next, we overexpressed RRM2 and its homolog RRM2B in THP1 cells, and meanwhile, decreased these two proteins in U937 cells. The knockdown of RRM2 and RRM2B was modest because they are pivotal for cell viability. Additional expression of RRM2 and RRM2B in THP1 cells did not enhance cell resistance to HU, even more vulnerable (Fig. 2c, d, Supplementary Fig. S2b). Furthermore, almost constant sensitivity was observed between control and RRM2 or RRM2B-knockdown U937 cells (Fig. 2e, f, Supplementary Fig. S2c).

We also detected TNFα that was potentially produced with HU treatment [30]. As shown in (Fig. 2g), a pretty high concentration of TNFα was detected in the supernatant of THP1 and NOMO1 cells. This was consistent with the necroptosis phenomenon of MLL-r AML cells. Taken together, we speculated that RRM2 inhibition was not the main factor for the selective killing of MLL-AF9 AML cells by HU treatment.

### HU target the defective MMP2 in MLL-AF9 leukemia cells

**Fig. 3:**
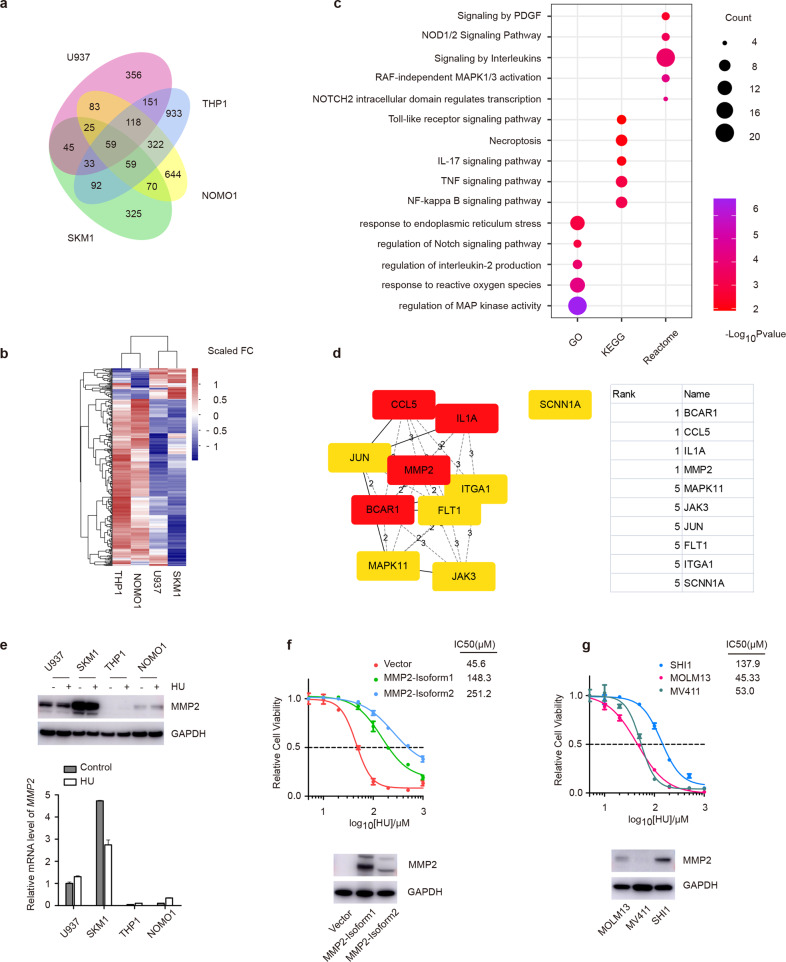
HU induces cell killing in MLL-r leukemia cells by MMP2 inhibition. **a** Venn diagram shows the non-overlapped and overlapped DEGs of cells with treatment of 100 μM HU after 24 h. **b**, **c** Heatmap (**b**) and GO, KEGG and Reactome analysis (**c**) of the DEGs shared in THP1 and NOMO1 cells. **d** 322 DEGs were taken to predict important nodes by cytoHubba, a plugin of Cytoscape, using the DNMC algorithm. **e** Gene expression and immunoblots of MMP2 in the indicated non-MLL and MLL-r leukemia cell lines. **f** THP1 cells were infected with the RRM2 and RRM2B lentiviral vectors. The indicated immunoblots and cell viability were analyzed. The IC_50_ of different cells was quantified. **g** Immunoblots of MMP2 in other three MLL-r AML cell lines, and their cell viability with 100 μM HU treatment after 72 h was measured. The IC_50_ of different cells was quantified.

To understand the mechanism HU treatment, we employed RNA sequencing (RNA-Seq) and mass spectrometry (MS) to systematically contrast the gene expression profiling and proteome of MLL-AF9 and non-MLL AML cells. Attention was paid to the genes whose expression exclusively changed in MLL-AF9 cells. The results showed THP1 and NOMO1 cells shared 322 differential expressed genes (DEGs), of which hierarchical clustering revealed a practically up-regulated gene expression pattern in these HU-sensitive cells (Fig. 3a, b). Next, we performed enrichment analysis through three annotation database and observed these DEGs principally involved in necroptosis-related inflammatory signals, ROS and endoplasm reticulum (ER) stress response (Fig. 3c).

The DEGs were analyzed using the Search Tool for the Retrieval of Interacting Genes/Proteins database to create a protein interaction network and seek hub genes (Fig. 3d). Interestingly, the *MMP2* gene (encoding Matrix Metallopeptidase 2) was a potential HU target and up-regulated both in THP1 and NOMO1 cells after treatment [31–33]. Furthermore, quantitative real-time PCR (qRT-PCR) and immunoblots results indicate a remarkably low level of MMP2 in THP1 and NOMO1 cells (Fig. 3e). Unlike RRM2, MMP2 was not up-regulated after HU treatment. Missing values of proteomic data were filtered out, and the 43 common differential proteins of THP1 and NOMO1 cells were mainly implicated in stress granule formation and translation regulation, followed by Gene Ontology annotation (Supplementary Fig. S3a, b).

To clarify MMP2 inhibition is responsible for HU-induced cell killing, we respectively overexpressed two different transcripts of the *MMP2* gene in THP1 cells. Expectedly, both the isoforms of MMP2 could increase the HU resistance of THP1 cells (Fig. 3f). Not only that, an MLL-r AML cell line SHI1 (MLL-AF6), in which MMP2 was highly expressed, displayed tolerance to HU compared with low MMP2 expressed MOLM13 (MLL-AF9) and MV411 (MLL-AF4) cell lines (Fig. 3g). Altogether, these presented results confirm that HU targeting insufficient MMP2 played a causal role in evoking HU susceptibility of MLL-r leukemia cells.

### Transcription of *MMP2* is directly controlled by MLL

Subsequently, we ought to unravel why these HU-sensitive MLL-r leukemia cell lines have reduced the expression of MMP2. Both DNA methylation and histone modification can control the expression of *MMP* genes [34]. Intriguingly, two studies found that H3K4 trimethyltransferase complex was recruited on the *MMP9* promoter in T-cell lymphoma cells, and knockdown of MLL showed reduced MMP2 and MMP9 in melanoma cells [35, 36], which enabled us to suspect that reduced MLL and H3K4 trimethylation was responsible for low expression of MMP2 in cell lines with MLL translocation.

**Fig. 4:**
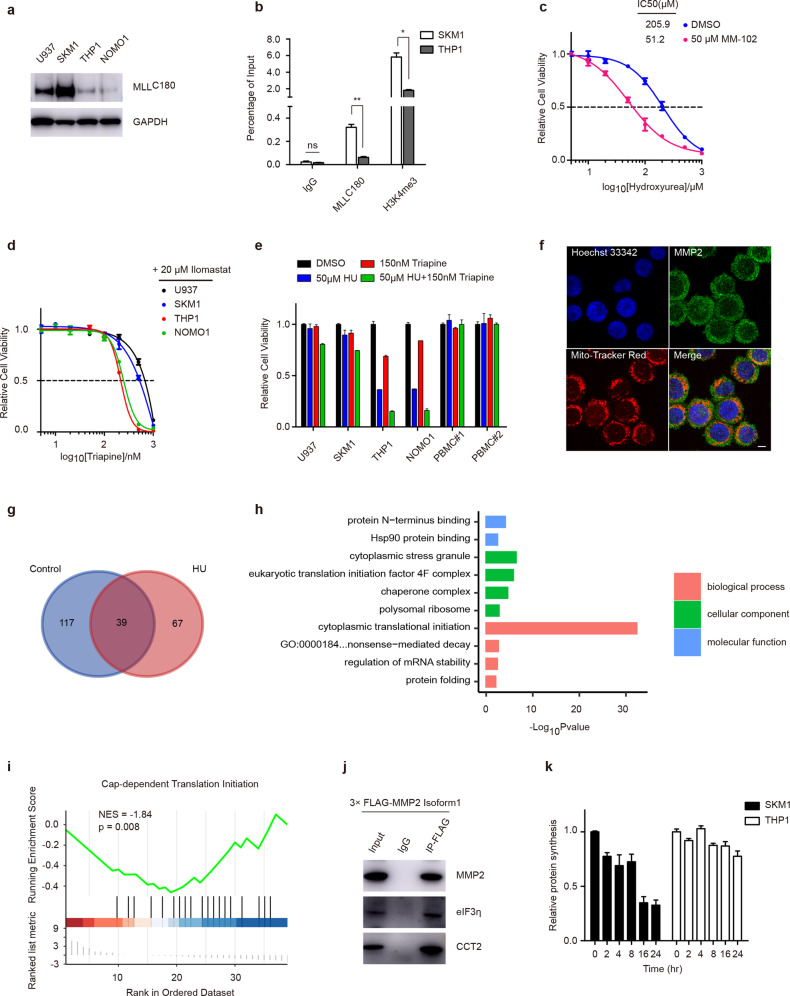
MMP2 is directly regulated by MLL and interacts with eIF3. **a** Immunoblots of MLL (MLL^C180^) in the indicated cell lines. **b** ChIP analyses at the promoter region of the MMP2 locus in the SKM1 and THP1 cells. *P < 0.05; **P < 0.01; the ns indicate no significant difference; two-tailed *t-*test. Data represent the means of triplicate reactions ± SD. **c** Cell viability of SKM1 was measured after 72-hour exposure to HU with co-treatment of 50 µM MM-102. **d** Cell viability was measured after 72-h exposure to triapine with co-treatment of 20 µM ilomastat. The IC_50_ of different cells was quantified. **e** Cell viability of indicated cells with co-treatment of 50 µM HU and 150 nM triapine after 72 h. **f** immunofluorescence visualized the localization of endogenous MMP2 in SKM1 cells. Scale bar, 5 μm. **g** Venn diagram shows the number of candidates who interacted with MMP2 in SKM1 cells without or with the treatment of 100 μM HU after 24 h. **h**, **i** GO enrichment (**h**), and GSEA analysis (**i**) of the common 39 interaction candidates of MMP2 in SKM1 cells with or without HU treatment. **j** THP1 cells were transfected with full-length MMP2, with a 3× FLAG-tag. Cell fractions were prepared for the Co-IP assays using the indicated antibodies. **k** Relative protein synthesis of SKM1 and THP1 cells with HU treatment after the indicated hours.

We observed that *MMP2* CpG sites were even unmethylated in most cell lines, suggesting that DNA methylation was not main factor for low MMP2 expression in MLL-r cells (Supplementary Fig. S4a). MLL C[^180^], representing WT MLL, was highly expressed in non-MLL leukemia cells (Fig. 4a). Besides, the MLL-AF6 leukemia cell line, SHI1, showed a more elevated MLL expression in comparison to HU-sensitive cells (Supplementary Fig. S4b). Chromatin immunoprecipitation (ChIP) assays were performed to verify that MLL directly regulated *MMP2* expression. Expectedly, MLL localized at the promoter region of *MMP2* locus, and THP1 cells displayed significantly less *MMP2* promoter occupancy of both MLL and H3K4me3 antibody than SKM1 cells (Fig. 4b). Moreover, we used MM-102, an inhibitor of MLL H3K4 trimethyltransferase, to treat SKM1 and examined whether this drug could repress MMP2 expression. The results revealed a decremental MMP2 level after MM-102 treatment, and the inhibitor also reduced the resistance of SKM1 cells to HU treatment (Fig. 4c, Supplementary Fig. S4c, d).

After unveil the relation among HU, MMP2, and MLL, cells were treated with MMP2 inhibitor ilomastat. However, THP1 and NOMO1 cells were not susceptible to this agent (Supplementary Fig. S4e). Considering HU is also an iron chelator [29], we speculated that MMP2 inhibition was sufficient but unnecessary for killing MLL-r cells, and combined ilomastat with triapine. Interestingly, co-treatment displayed selective killing in THP1 and NOMO1 cells (Fig. 4d), implying that MMP2 might be a support factor for iron chelation-induced cell stress. These two cell lines were also vulnerable to the combination of HU and triapine, without toxicity to healthy human peripheral blood mononuclear cells (Fig. 4e, Supplementary Fig. S4f).

To preliminarily understand MMP2 function, we conducted immunofluorescent assays on SKM1 cells and observed that most MMP2 localized at cytoplasm, although studies have demonstrated their findings in mitochondria and nucleus [37] (Fig. 4f). Next, co-immunoprecipitation (Co-IP) and MS were combined to investigate proteins interacting with MMP2. We collected the intersection of 156 candidates from the control group and 106 candidates from HU treated group and performed analysis using Gene Ontology and Gene Set Enrichment Analysis methods. These 39 proteins are mainly implicated in translation, nonsense-mediated decay, and unfolded protein response (UPR), and proteins related to translation initiation reduced after HU treatment (Fig. 4g–i). The interplays of MMP2 with eIF3 and chaperone protein were further confirmed in THP1 cells (Fig. 4j). In addition, after treated with HU for a few hours, SKM1 cells displayed reduced protein synthesis compared with THP1 cells (Fig. 4k). Altogether, we validated that MLL controls the transcription of *the MMP2* gene, and decreased MLL in MLL-r leukemia cells accounts for insufficient MMP2 and HU sensitivity, and our rough exploration also suggests a possible role of MMP2 in UPR pathways.

### Prognosis of MMP2 expression in HU administrated AML patients

**Fig. 5:**
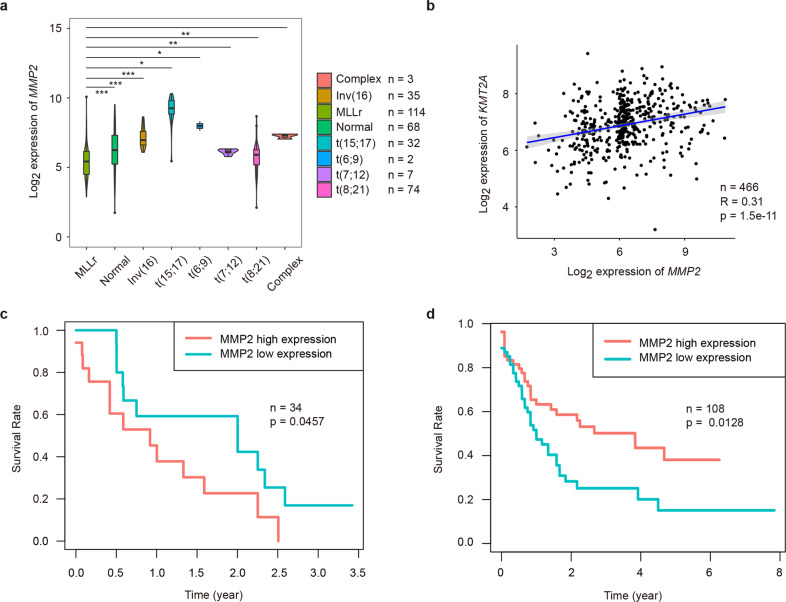
Low expression of MMP2 indicates a better prognosis in AML patients with HU treatment. **a** MMP2 expression levels in primary AML patient samples carrying indicated mutations were shown by analyzing the publicly available microarray datasets. Wilcoxon rank-sum tests were performed. Sample size and P-values were presented. * for *P* < 0.05, ** for *P* < 0.01, *** for *P* < 0.001. **b** Co-expression correlations between MMP2 and KMT2A (MLL). **c** Kaplan-Meier survival curve of AML patients with HU treatment from the TCGA dataset (low expression: 50%, *n* = 17; high expression: 50%, *n* = 17). **d** Kaplan-Meier survival curve of AML patients without HU treatment (low expression: 50%, *n* = 54; high expression: 50%, *n* = 54).

To elucidate the clinical significance of MLL and MMP2 expression, we investigated the co-expression relationships between *KMT2A* (encoding MLL) and *MMP2* genes using available microarray-based gene expression profiling of AML patients, and also examined the association between MMP2 expression and patient survival with HU treatment using the TCGA AML dataset. Samples less than two replicates and unknown karyotypes were screened out, and the results show that patients designated with MLL translocation have a significantly reduced expression level of *MMP2* compared with other mutations (Fig. 5a). Furthermore, the correlation coefficient also indicates the positive correlation between *KMT2A* and *MMP2* gene expression (Fig. 5b). In survival analysis, we selected patients following chemotherapy (HU dosage > 0) and observed that low *MMP2* expression revealed a better prognosis outcome (Fig. 5c). In addition, we collected the remaining data (HU dosage = 0 and HU information not available), trying to reduce the impact of HU and analyze the effect of MMP2 expression in survival without therapeutic interventions. Interestingly, low MMP2 level suggested a shorter life term in these patients, consistent with high malignancy of MLL-r leukemia (Fig. 5d). Altogether, clinical evidence strongly supports that the reduction of MLL and MMP2 are significantly related to the acquisition of HU sensitivity in MLL-r AML cells.

## Discussion

Oncogene-induced epigenetic changes occurring in hematopoietic progenitors lead to tumorigenesis and alter drug sensitivity in the different subtypes of leukemia [38]. Many pieces of research have denoted that histone modification was closely associated with drug effect, and lack of H3K4 trimethylation can either cause drug resistance, by multiple factors such as cell cycle defect [39] and less incorrect replication fork formation [40], or sensitivity through down-regulating the targets of drug [41]. Most drugs, even FDA-approved, generate off-target toxicity because complicated cellular environments can promote small molecules promiscuously interacting with unanticipated proteins [42]. However, this molecular promiscuity of lead compounds may help assess the safety of novel drugs and offer new strategies for drug repurposing [43]. Compared with drugs targeting a single biological entity, multi-target approaches provided higher efficacy and less drug resistance [44]. Although numerous studies set HU as RRM2 inhibitors and representative DNA damage agents, indeed, it should be not forgotten that HU has potential metalloenzyme targets, and effective mechanisms of oxidative stress are not yet well-understood [45]. HU contains a functional group of hydroxamic acid, usually utilized to design histone deacetylases and MMPs inhibitors [33].

In many cases, HU treatment for DNA damage induction was in millimolar concentration and short-term [17, 18, 46, 47], different from the long-time treatment under moderate dose in our study. DNA synthesis instantaneously decayed upon HU treatment but rose again in step with CHK1-induced RRM2 accumulation to counteract inhibition [27, 48], during which DNA damage was not significantly increased. When under high dose HU treatment, cells cannot afford this pressure and result in fork stalling. MMP2 is not up-regulated with HU treatment, and this might convert MMP2 to be the principal target in modest concentration. A study has demonstrated that HU could induce cell death not by RRM2 inhibition and dNTPs depletion [49]. Intriguingly, MLL-r leukemia cells treated with 100 μM HU underwent necroptosis, for a typical feature of cell swelling, while in the condition of 10 mM HU they were through the apoptotic process because of cell shrinkage and DNA fragmentation (Fig. 2c, Supplementary Fig. S5a, b). Distinct cell death phenotypes depend on the different drug doses [50, 51], and this might be owing to drug off-target effects.

Furthermore, MMP2 inhibition cannot be the unique factor for the selective killing of MLL-r AML cells, and the iron chelation induced cell stress following HU treatment is essential. MMPs are classically considered enzymes responsible for the degradation of extracellular matrix proteins [37]. Recent studies showed multiple intracellular functions of MMPs with their new substrates. Most MMPs contain nuclear localization signalling sequence in the catalytic domain, allowing them to enter the nucleus, and this will induce apoptosis by cleaving PARP, an import component of the DNA repair complex [52, 53]. Nuclear MMPs bind to the promoter of genes associated with viral immunity and cleave the protein product, regulating at transcriptional and post-transcriptional levels [37]. Studies of cytoplasmic and mitochondrial MMPs still blossomed, where MMP2 can be directly activated by oxidative stress, and catalyze proteolysis of protein kinase, Ca^2+^-ATPase, and mitochondrial Hsp60, thus to involve in ER signals, and mitochondria dysfunction [54]. Our tentative investigation indicated a novel interaction of MMP2 with the eIF3 protein, which probably facilitated MMP2 to mediate nonsense-mediated mRNA decay under innocuous ER stress. The pathway enhances the threshold for triggering ER stress-induced UPR and prevents cell death [55], and the balance might be disrupted by MMP2 inhibition. Enrichment of ER stress response, and stress granule formation in gene expression and protein profilings support this speculation.

A very suggestive study identified a novel inhibitor, CCI-006, which targeted mitochondrial respiration and displayed cytotoxicity against MLL-r leukemia cells, while the selective killing phenotype was unsuccessfully replicated using other mitochondrial stressors [8]. The compound harboured a sulfonamide group and was conceived as a potential carbonic anhydrases inhibitor [8]. Interestingly, this structure is applied to design MMPs inhibitors as well [56]. Nevertheless, the exact mechanism of how MMP2 regulates the UPR pathway should be furtherly illustrated. Most results were obtained from immortalized cell lines, and it is imperative to validate the effectiveness of HU in patient-derived MLL-r AML xenograft models before clinical practice.

In conclusion, we firstly demonstrated that HU selectively kills MLL-r AML cells by MMP2 inhibition. H3K4me3 activates the *MMP2* expression, which is repressed in MLL-r cells due to the defective WT MLL. MMP2 supports cell viability through prohibiting the activation of UPR pathway under low level of ER stress. Our findings reveal a new cell killing mechanism of HU in MLL-r AML cell lines and this might assist HU to apply in other cancers.

## Materials and methods

### Reagents and cell culture

Bortezomib, cytarabine, ilomastat, MM-102 (Selleck Chemicals, Houston, TX, USA). HU, osalmid, triapine (TargetMol, Shanghai, China). Most AML cell lines were gifts of Prof. James Hsieh (MSKCC, New York, USA). SKM1 (JCRB cell bank, Osaka, Japan). SHI1 was provided by the Cyrus Tang Hematology Center (Soochow University, Suzhou, China). Human PBMCs were collected from two healthy individuals. All cells were cultured in RPMI-1640 (HyClone, UT, Logan, USA) containing 10% FBS (Gibco, Carlsbad, CA, USA) at 37 °C with 5% CO_2_.

### Cell survival viability, apoptosis, and cell cycle assays

Cell viability was analyzed using the CCK8 (TargetMol). The apoptosis and cell cycle were performed using the Annexin V-PE Apoptosis Detection Kit and APC BrdU Flow Kit (BD Pharmingen, San Diego, CA, USA). Data produced by the flow cytometer were analyzed using the FlowJo software.

### Mitochondria membrane potential and TNFα measurement

Mitochondria membrane potential assay was conducted using JC-1 fluorescence probe (Beyotime, Shanghai, China). TNFα was detected using Human TNFα ELISA Kit (Beyotime).

### Immunoblots

Rabbit antibodies against human RRM1, RRM2, RRM2B, CCT2 (ABclonal Technology, Wuhan, China). Rabbit antibodies against human GAPDH, ATR, Phospho-ATR/S428, ATM, Phospho-ATM/S1981, CHK1, Phospho-CHK1/S345, CHK2, Phospho-CHK2/T68, γH2A.X/S139, MMP2, MLL^C180^ and H3K4me3, and rat antibody against human RPA32 (Cell Signaling Technology, Danvers, MA, USA). Rabbit antibody against human Phospho-RPA32/S4S8 (Bethyl Laboratories, Montgomery, TX, USA). Mouse antibody against human eIF3η (Santa Cruz, CA, USA).

### Lentivirus-based knockdown and overexpression

For knockdown, target sequences against human RRM2 and RRM2B and a scramble sequence were inserted into the pLKO.1 mcherry vector (Addgene, Watertown, MA, USA). For exogenous expression of MMP2, the entire length (isoform1) and N-terminal truncated (isoform2) cDNA were inserted into the pCDH-copGFP (Addgene) vector. Viral particles were produced by transfection of vectors into HEK-293 T cells combined with helper plasmids using ViaFect™ Transfection Reagent (Promega, Madison, WI, USA). 24 h later, the supernatant was harvested and used to transduce cells, followed by spinoculation. Positive cells were FACS-purified based on the fluorescent reporter protein. All sequences are available in the Supplementary Table S1.

### DNA methylation detection and qRT-PCR

Genomic DNA was purified using a commercial DNA extraction kit (TIANGEN, Beijing, China). Samples were then treated with sodium bisulphate to convert unmethylated cytosine to uracil using an EZ DNA Methylation Kit (Zymo Research, Irvine, CA, US). The bisulphite transformed DNA was used to amplify the product in the MMP2 promoter area, and the level of MMP2 methylation level was represented by the percentage of methylated CpG in methylated unmethylated CpG using qRT-PCR assays.

For qRT-PCR, RNA was isolated with RNAprep pure Cell/Bacteria Kit (TIANGEN) and reverse-transcribed with the GoScript™ Reverse Transcription System (Promega). The collected cDNA and genomic DNA collected in other experiments were amplified using Eastep® RT Master Mix (Promega). The relative fold expression values were determined by the ΔΔCt method and normalized to GAPDH as a reference gene. Primers are available in Supplementary Table S1.

### RNA sequencing and protein identification by MS

The mRNA-Seq library was constructed and sequenced using the Illumina TruSeq library construction kit and BGISEQ-500 PE100 (BGI Technology Service, Wuhan, China). Differential expressed genes were selected based on |log_2_FoldChange | > 1 and *P*-value < 0.05. Bioinformatic analysis was performed using the R package ‘clusterProfiller’.

Protein samples were resolved by SDS-PAGE and visualized using SilverQuest^TM^ Silver Staining Kit (Thermo Fisher Scientific, Waltham, MA, USA). Gels were sent to the Public Technology Platform of Shanghai Jiaotong University School of Medicine and analyzed using label-free LC-MS. Differential proteins were selected based on |log_2_FoldChange | > 1 and -lgP > 0 (proteins have missing value were filtered out). Profiling results are available in Supplementary Tables S2 and S3.

### ChIP and Co-IP assays

ChIP assays were performed following the described protocol [11] using anti-MLL^C180^ and H3K4me3 antibodies. Primers used for ChIP-qPCR assay were located at the *MMP2* promoter region (−394 bp to −169 bp relative to TSS).

For Co-IP, cell lysis was incubated with an anti-MMP2 antibody and beads overnight. Beads were collected and washed 3 times, followed by denaturation in the SDS loading buffer. Candidates who interacted with MMP2 identified in MS were selected based on IP/IgG > 3 and values not missed in IP groups. See primer sequences and IP-MS results in Supplementary Table S4. Mouse anti-FLAG monoclonal antibody (Sigma-Aldrich, Saint Louis, MO, USA).

### Wright-Giemsa staining, immunofluorescence and protein synthesis assays

Cytospin preparations of 1 × 10^4^ cells were air-dry and incubated in 1:1 Wright-Giemsa solution/Phosphate buffer (Sangon Biotech, Shanghai, China) for 10 min. The slides were then washed in water and examined under microscopy.

For IF assays, cells were stained with Mito-Tracker Red (Beyotime) following recommendations before spin. Subsequent operations were performed following the described protocol [17].

Protein synthesis assays were performed using Click-iT Plus OPP Protein Synthesis Assay Kits (Thermo Fisher Scientific) and analyzed by flow cytometer.

### Data analysis of AML patients

Raw microarray datasets of primary AML patient samples were downloaded from Gene Expression Omnibus. The raw AML datasets used in this study were accessible under GSE14062, GSE19577, GSE35784, GSE17855, and GSE52891. The profiles were merged and normalized using the R package ‘limma’. With stronger signals, ‘201069_s_at’ and ‘212080_s_at’ were selected to represent MMP2 and MLL respectively. Samples of the same mutation with less than two replicates were deleted. Wilcoxon rank-sum tests were performed for the pairwise comparisons.

FPKM matrix and clinical information of AML patients were downloaded and integrated using the R package ‘TCGAbiolinks’. Gene expression and Kaplan-Meier curves of patients with or without the intervention of HU were evaluated. The TCGA datasets are available in Supplementary Tables S5 and S6.

### Statistical analysis

The two-tailed Student *t*-test and log-rank test were used to analyze the difference between the control and experimental group. The statistically significance level is indicated as * for *P* < 0.05, ** for *P* < 0.01, or *** for *P* < 0.001.

## Supplementary information

supplementary figures

Supplementary Tables

original western blots

## Data Availability

All data generated in our study or from other researches are included in this published article. Further information is available from corresponding author upon reasonable request.
