# Supplementary material for: Targeting matrix metallopeptidase 2 by hydroxyurea selectively kills acute myeloid mixed-lineage leukemia
Source: Cell Death Discov. 2022 Apr 8;8:180. doi: 10.1038/s41420-022-00989-4 (PMC8993889; doi:10.1038/s41420-022-00989-4)
Supplement: Supplementary file 1 — supplementary figures [file 41420_2022_989_MOESM1_ESM.docx]

1. **Supplementary figures**

**
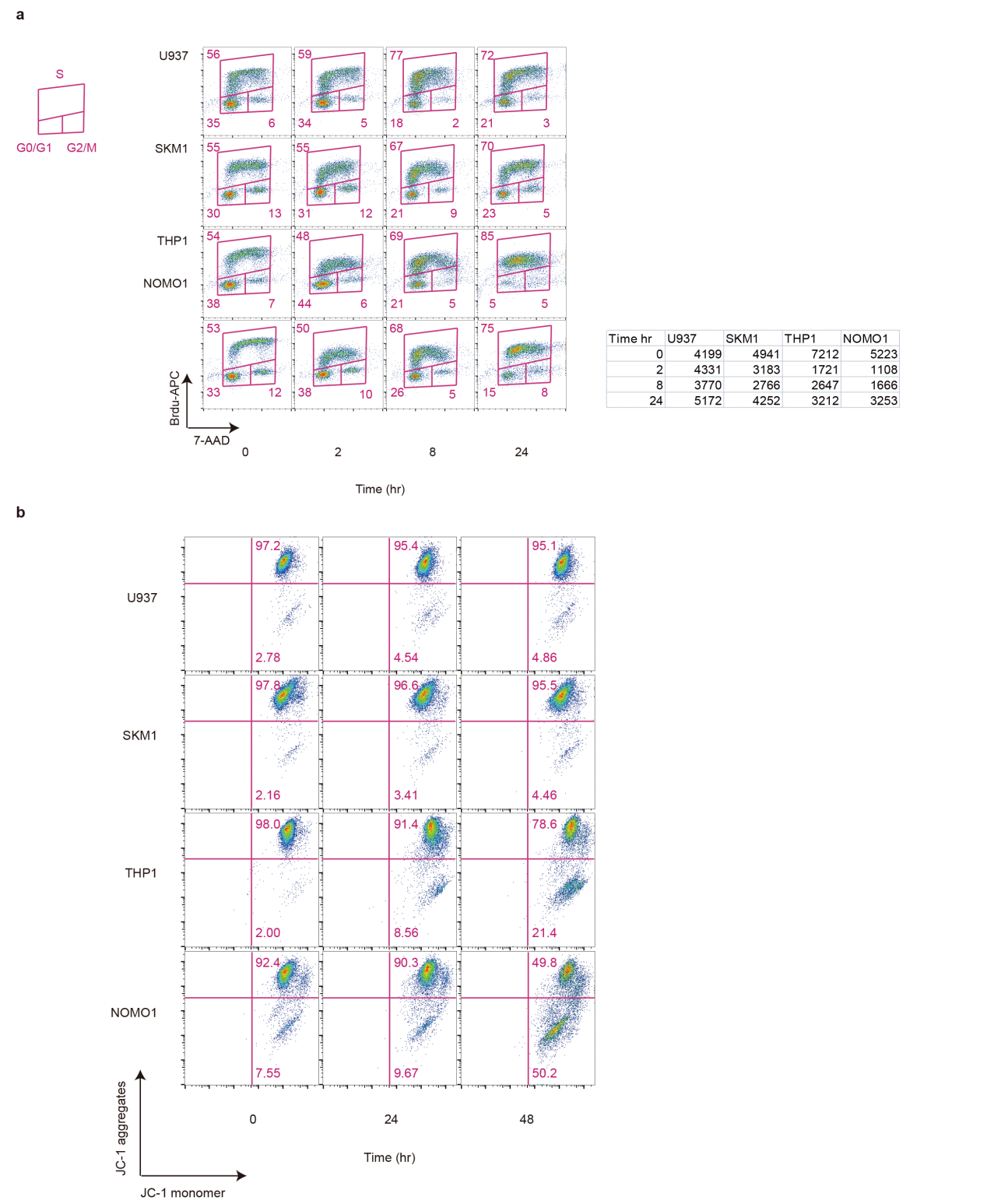
**

**Supplementary Fig. S1 HU induces cell cycle arrest and loss of mitochondrial membrane potential in MLL-AF9 AML cells.**

**a-b** The mean of BrdU incorporation in the S phase (**a**) and mitochondrial membrane potentials (**b**) of cells with the treatment of 100 μM HU after the indicated hours.
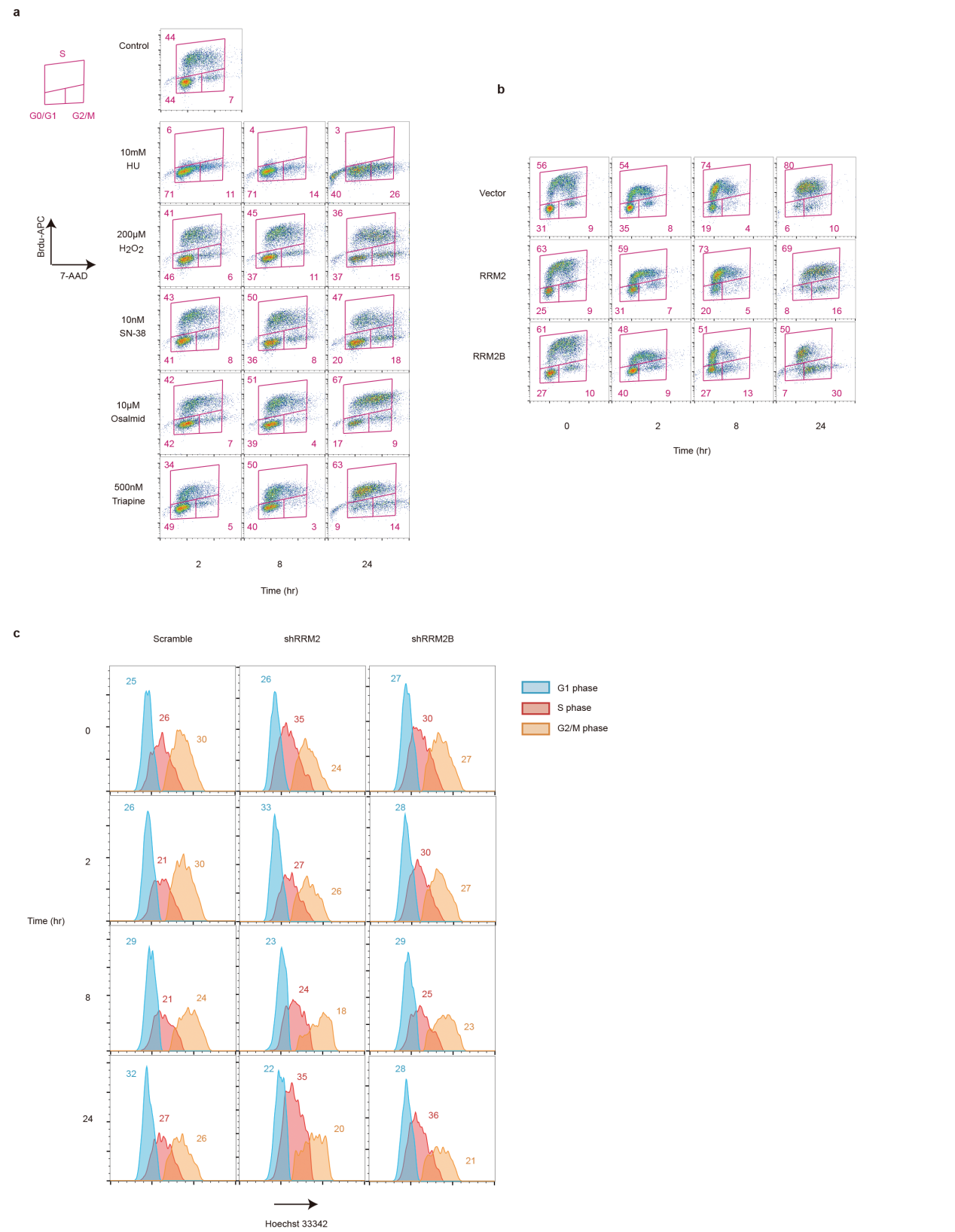


**Supplementary Fig.S2 DNA damage and RRM2 are not responsible for HU induced cell cycle arrest in MLL-AF9 AML cells.**

**a** Cell cycle profiling of the THP1 cells after treatment with the indicated DNA damage agents at 0, 2, 8, 24 hours. **b-c** THP1 (**b**) and U937 (**c**) cells were infected with the indicated lentiviral vectors. Cell cycle profiling of the indicated cells were analyzed.


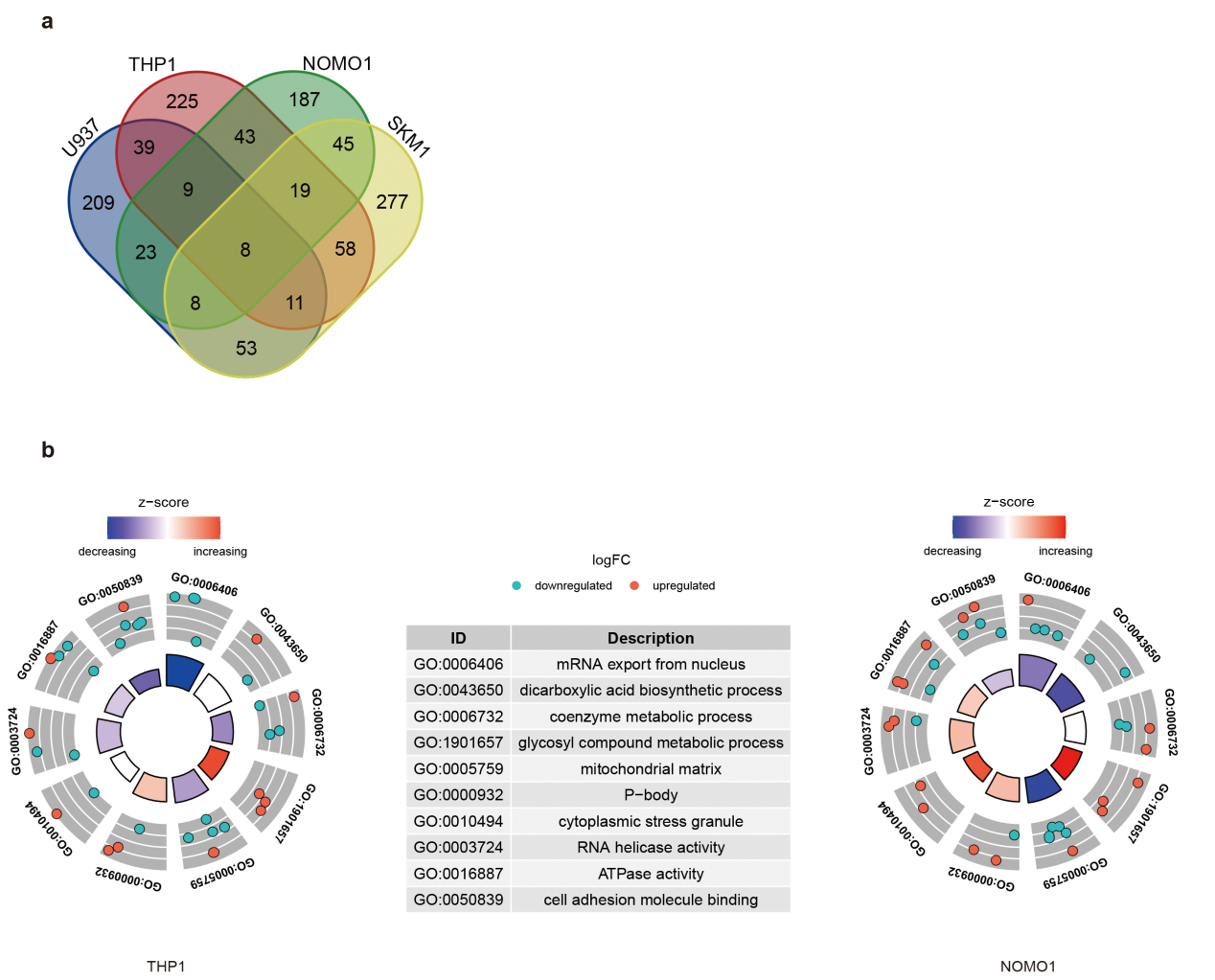


**Supplementary Fig.S3 Analysis of proteomic data.**

**a** Venn diagram shows the number of differential proteins in the indicated cell lines with the treatment of 100 μM HU after 24 hours. **b** GO enrichment analysis of 43 differential proteins shared in THP1 and NOMO1 cells.


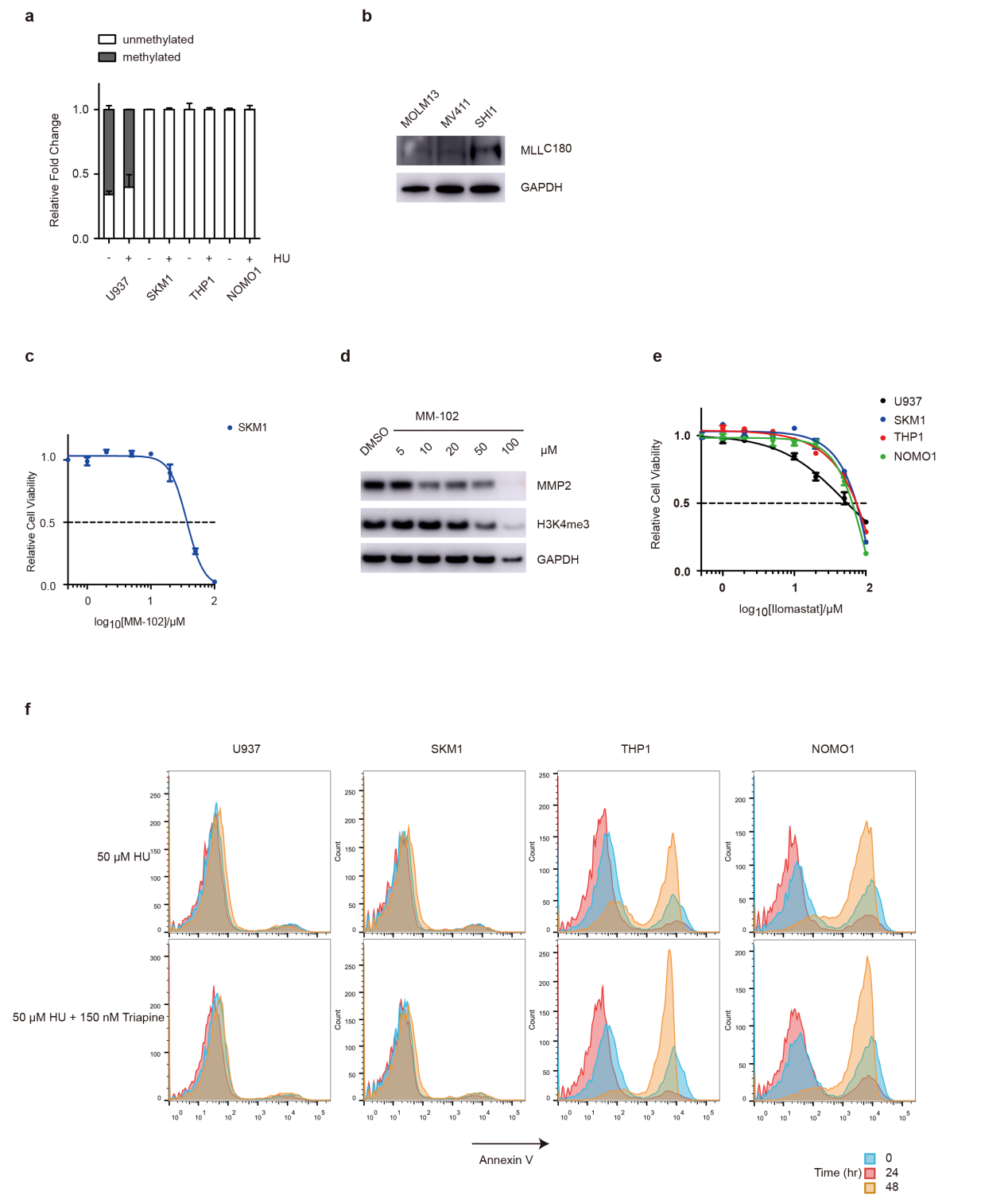


**Supplementary Fig.S4 Enhancement of MMP2 inhibition or iron chelation promotes cell killing of MLL-r cells.**

**a** The methylation status of *MMP2* promoter in cells with 100 μM HU treatment after 24 hours. **b** Immunoblots of MLL^C180^ in other MLL-r AML cell lines. **c** After 72-hour exposure to MM-102, the cell viability of SKM1 was measured. **d** Immunoblots of MMP2 and H3K4me3 in SKM1 cells with treatment of MM-102 at the indicated concentration after 24 hours. **e** Cell viability of ilomastat treatment after 72 hours. **f** Apoptosis of cells with co-treatment of 50 µM HU and 150 nM triapine after the indicated hours.


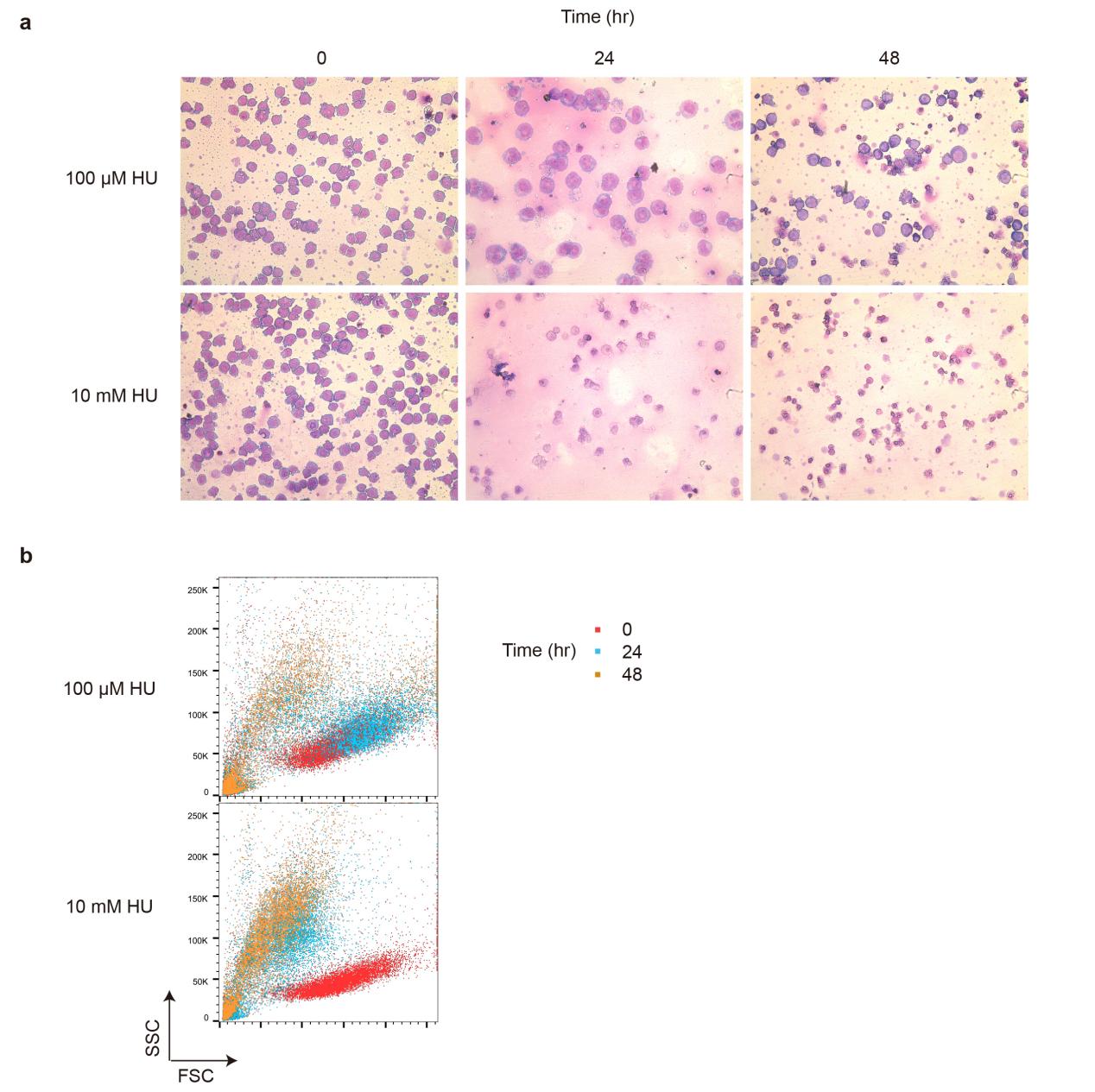


**Supplementary Fig.S5 Cell death phenotype under treatment of different HU dosage.**

**a-b** Wright-Giemsa staining and cell volume (**a**) analysis using flow cytometry **(b)** of THP1 cells with a modest and high dosage of HU after the indicated hours. Magnification is 200×.

1. **Supplementary tables**

**Supplementary Table S1 Primer sequences**

**Supplementary Table S2 RNA-Seq matrix of AML cells with HU treatment**

**Supplementary Table S3 MS data of AML cells with HU treatment**

**Supplementary Table S4 CoIP-MS assays data of SKM1 cells without or with HU treatment using CST MMP2 antibody**

**Supplementary Table S5 FPKM matrix of AML patient samples downloaded from TCGA database**

**Supplementary Table S6 Clinical informations of AML patient samples downloaded from TCGA database**
