# Supplementary material for: Targeting matrix metallopeptidase 2 by hydroxyurea selectively kills acute myeloid mixed-lineage leukemia
Source: Cell Death Discov. 2022 Apr 8;8:180. doi: 10.1038/s41420-022-00989-4 (PMC8993889; doi:10.1038/s41420-022-00989-4)
Supplement: Supplementary file 3 — original western blots [file 41420_2022_989_MOESM3_ESM.docx]

**Fig. 2a** AML cell lines at 0, 8, 24 hours with 100 μM HU treatment.


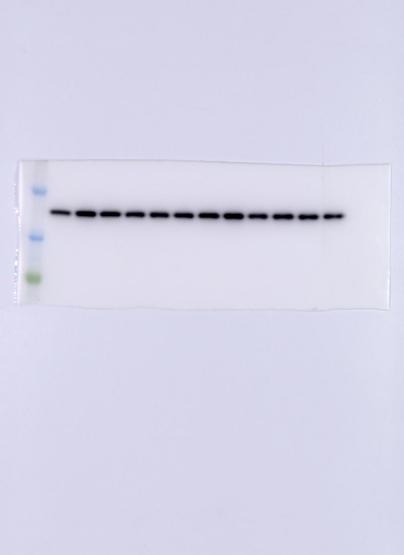

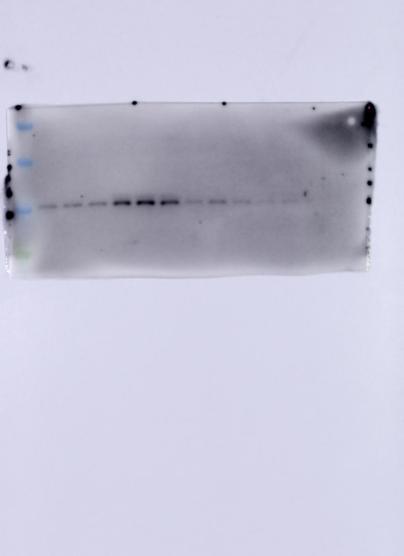

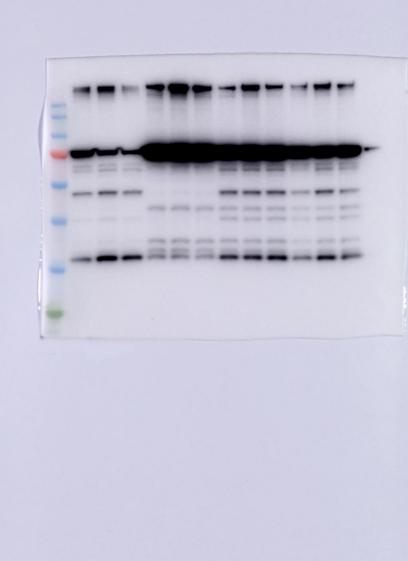


0

8

24

0

8

24

0

8

24

0

8

24

U937

SKM1

THP1

NOMO1

+HU (hr)

GAPDH RPA32 pRPA32 S4/S8


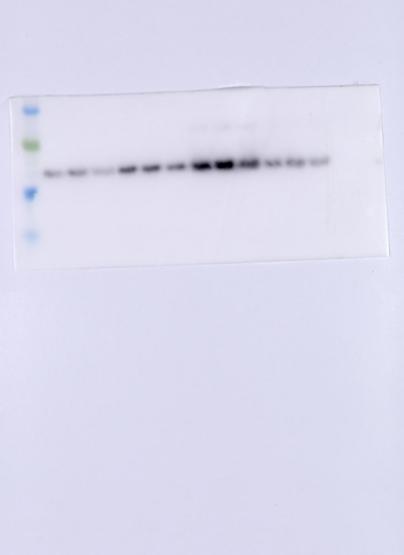

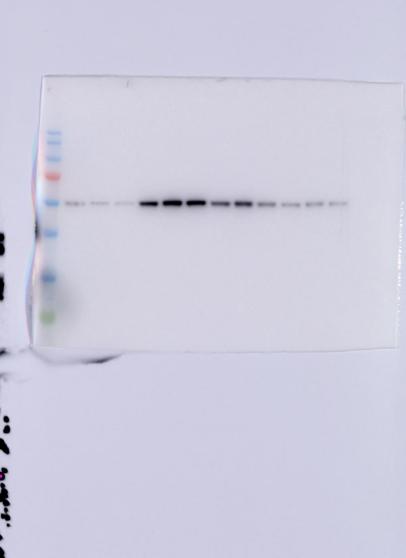

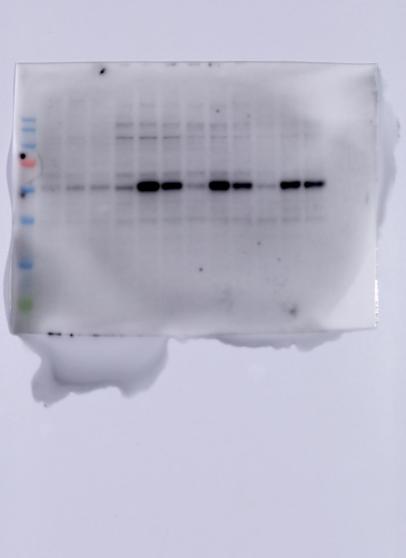


22kDa

170kDa

130kDa

93kDa

70kDa

53kDa

41kDa

30kDa

γH2A.X CHK1 pCHK1 S345


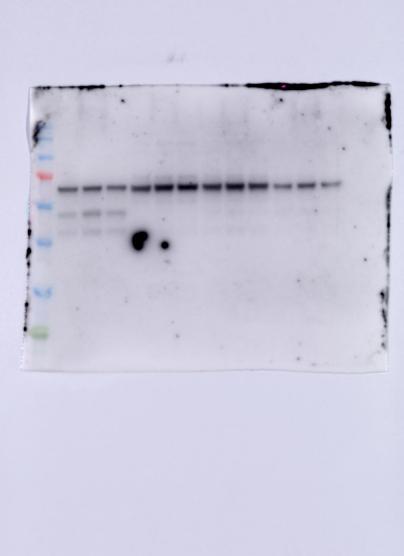

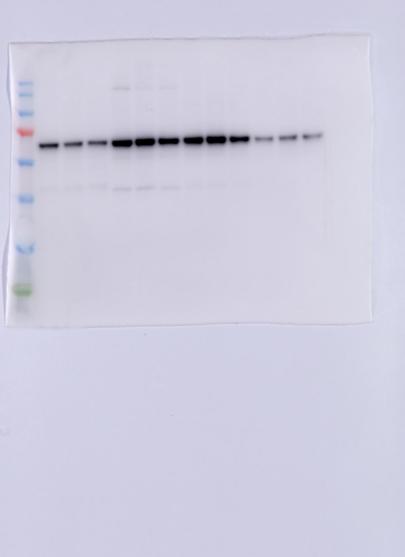

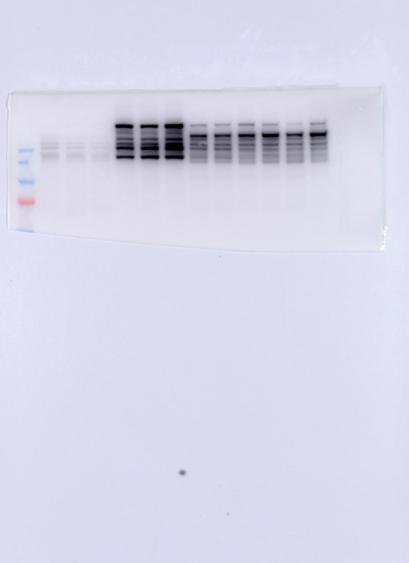


CHK2 pCHK2 T68 ATM


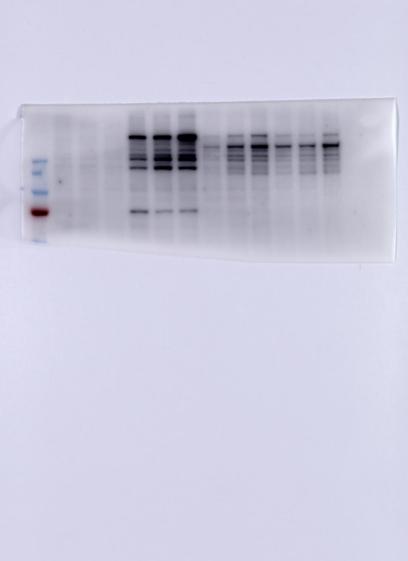

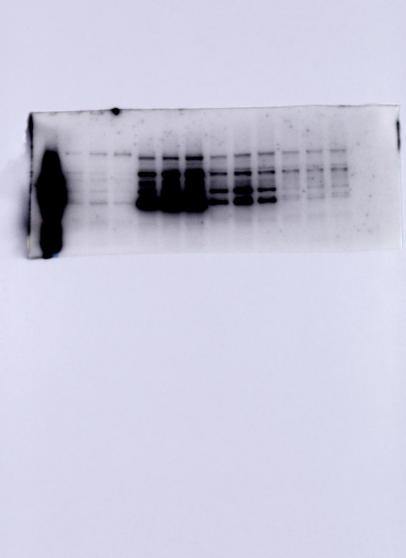

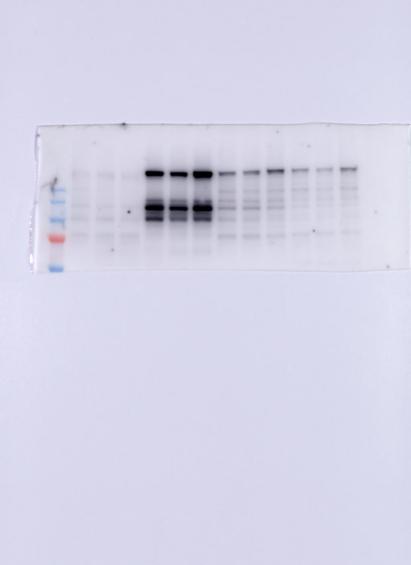


pATM S1981 ATR pATR S428


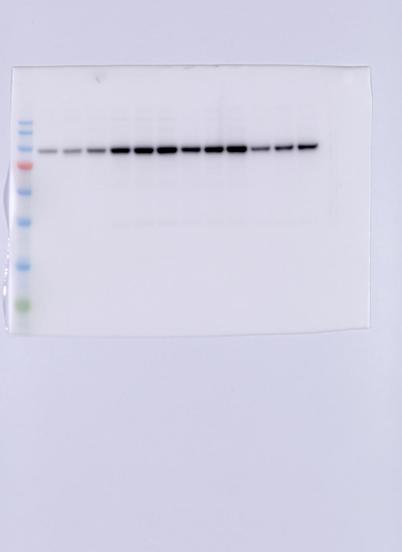

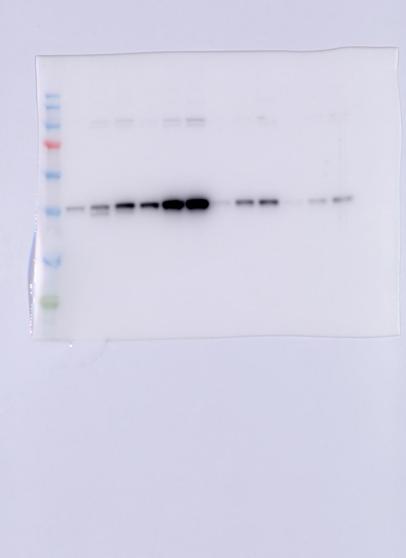

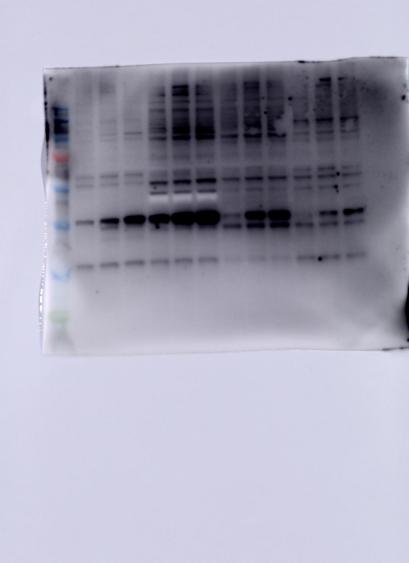


RRM1 RRM2 RRM2B

**Fig. 2c** THP1 cells were infected with the RRM2/RRM2B lentiviral vectors

-

+

-

+

-

+

HU

THP1

THP1-RRM2

THP1-RRM2B


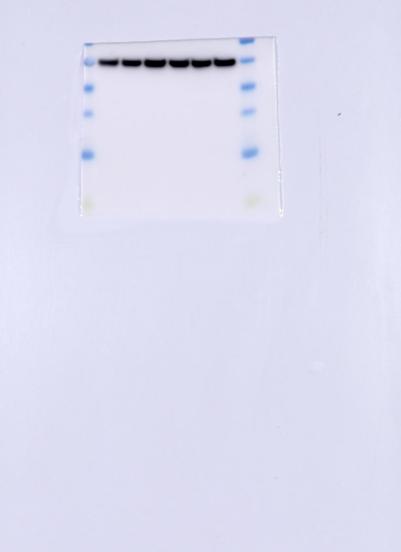

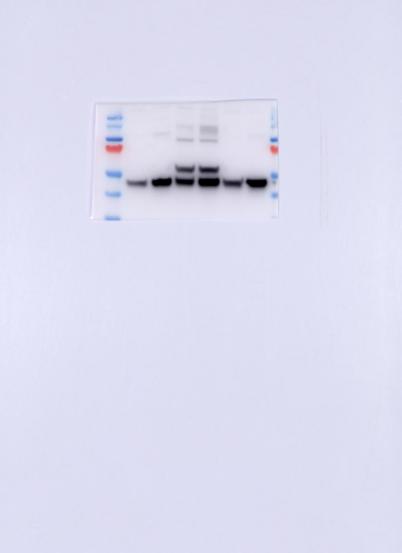

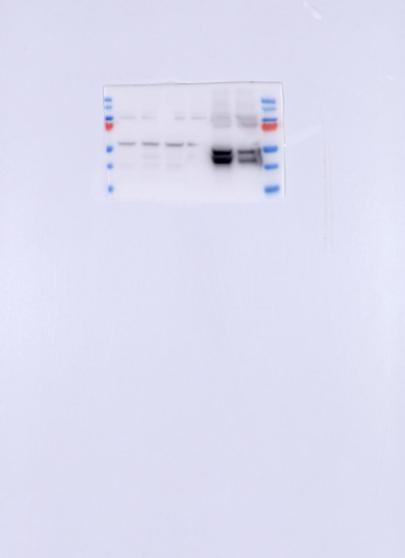


GAPDH RRM2-GFP(blue) RRM2B-GFP(blue)

RRM2 RRM2B


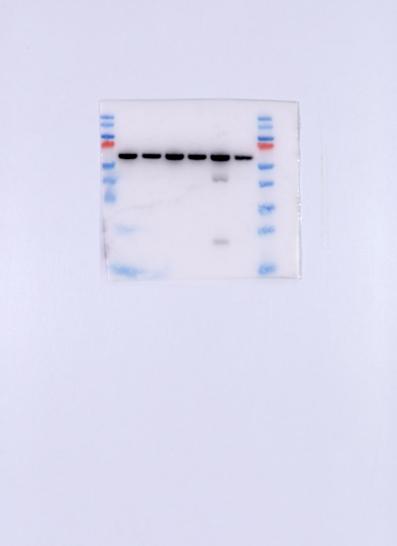

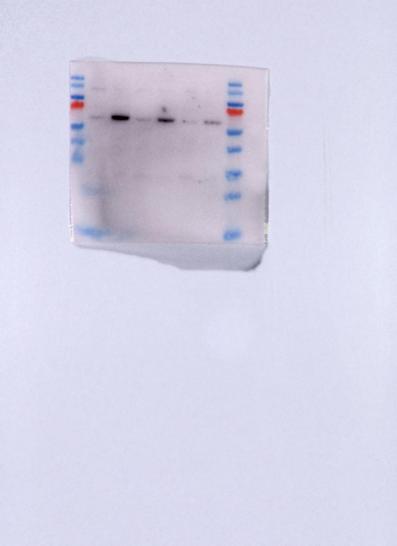

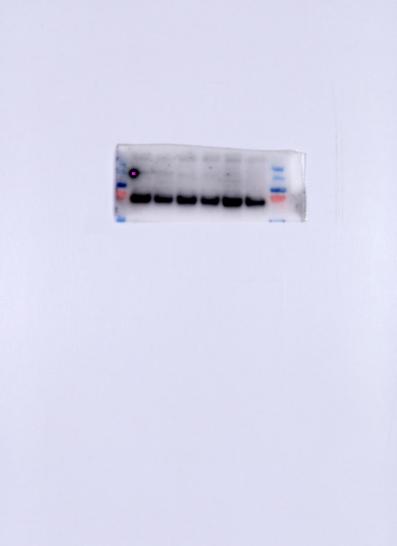


CHK1 pCHK1 S345 CHK2


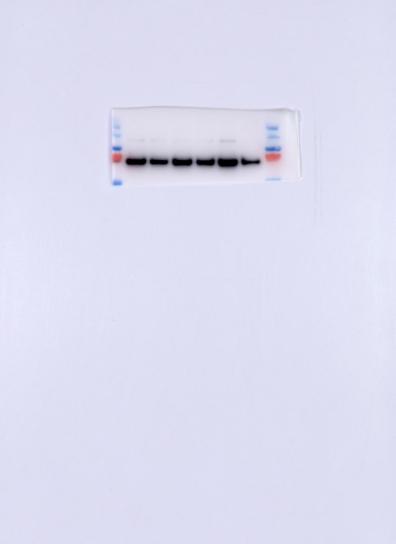

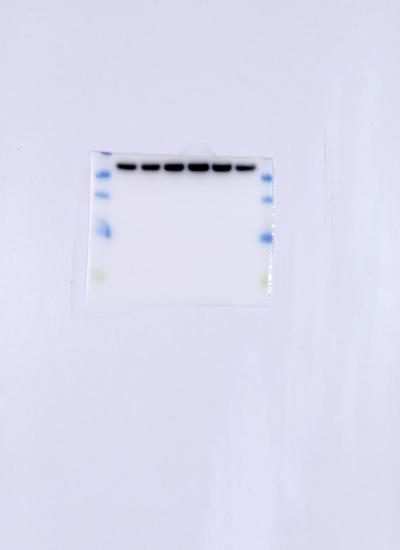

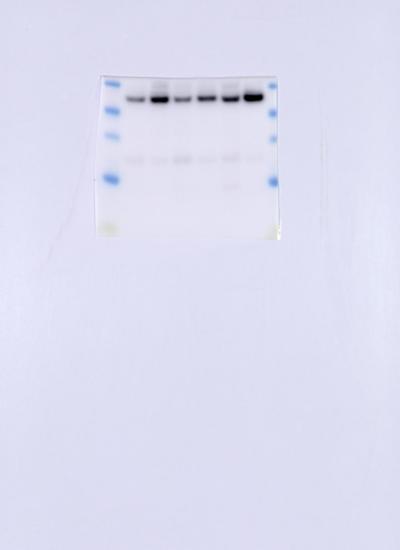


pCHK2 T68 RPA32 pRPA32 S4/S8


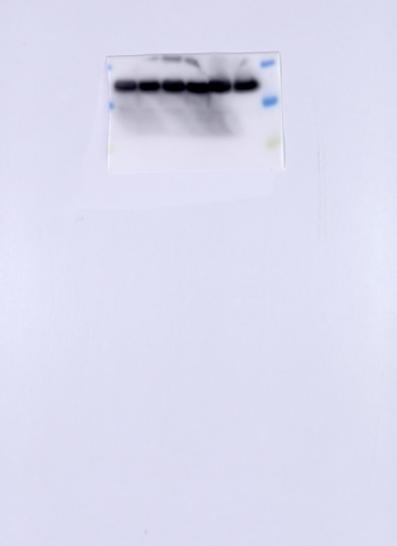


γH2A.X

**Fig. 2e** Knockdown of RRM2 or RRM2B was performed in U937 cells.

Scramble

shRRM2B


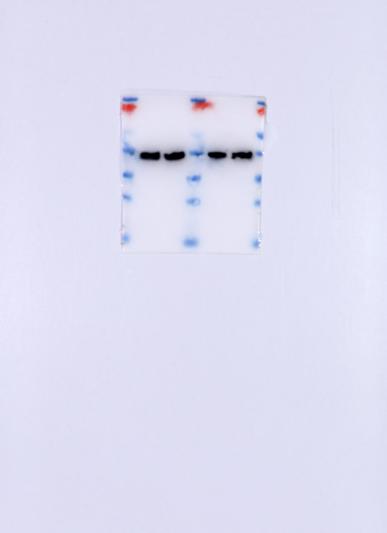

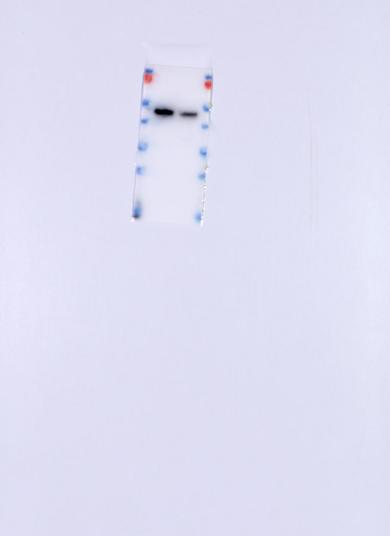

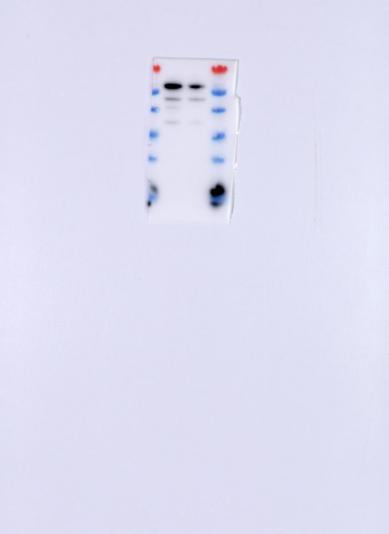


shRRM2

Scramble

Scramble

shRRM2B

shRRM2

Scramble

GAPDH RRM2 RRM2B

**Figure 3e** MMP2 in non-MLL and MLL-r leukemia cell lines with HU treatment.


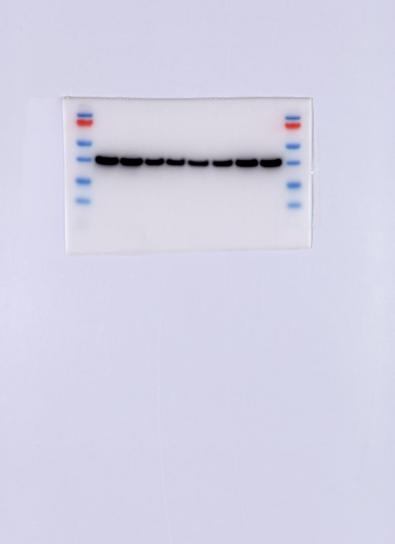

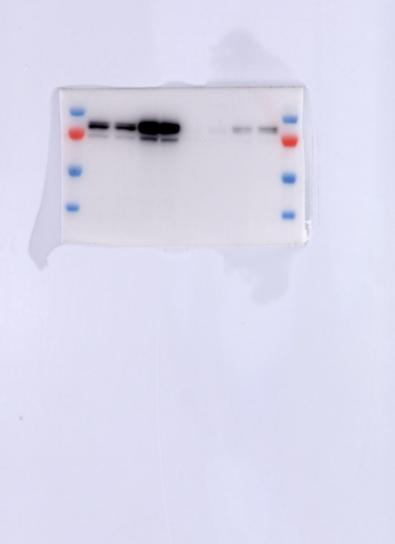


-

+

-

+

-

+

-

+

U937

SKM1

THP1

NOMO1

HU

GAPDH MMP2

**Fig. 3f** THP1 cells were infected with the RRM2 and RRM2B lentiviral vectors.


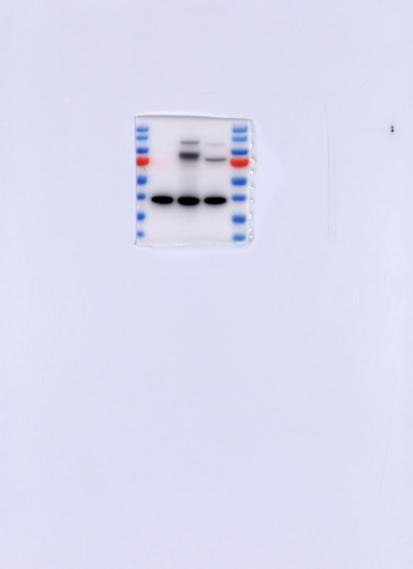

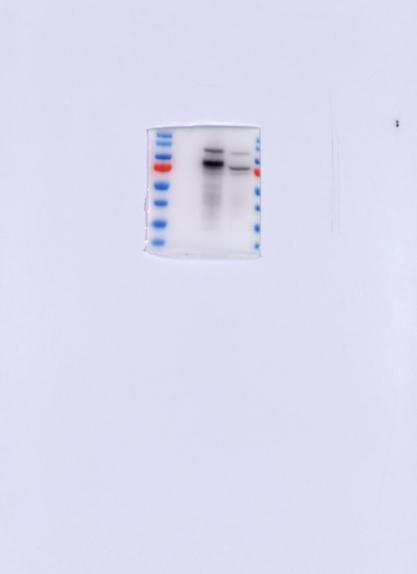


Vector

MMP2 isoform1

MMP2 isoform2

GAPDH MMP2-GFP(blue)

MMP2

**Fig. 3g** Immunoblots of MMP2 in other three MLL-r AML cell lines.


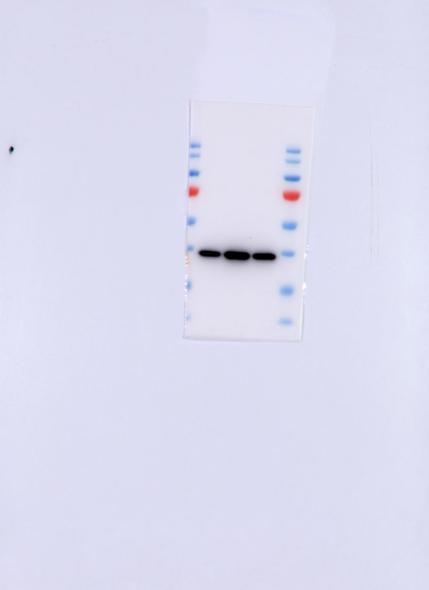

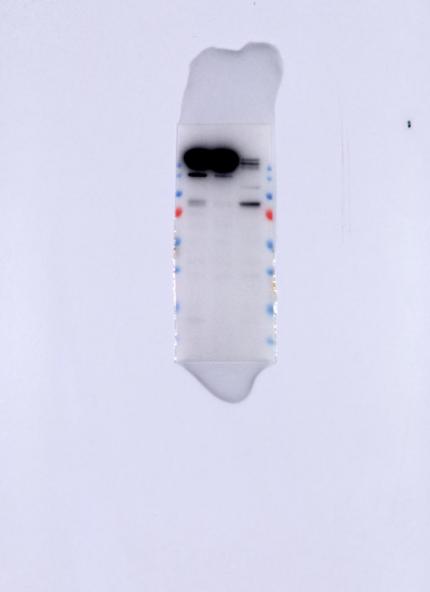


MOLM13

MV411

SHI1

GAPDH MMP2

**Fig. 4a** Immunoblots of MLL (MLL^C180^) in AML cell lines.


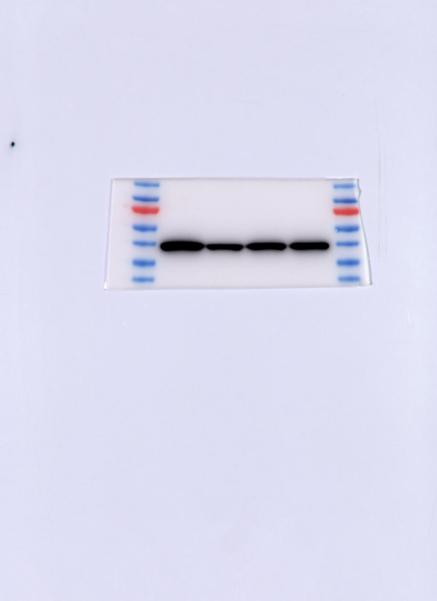

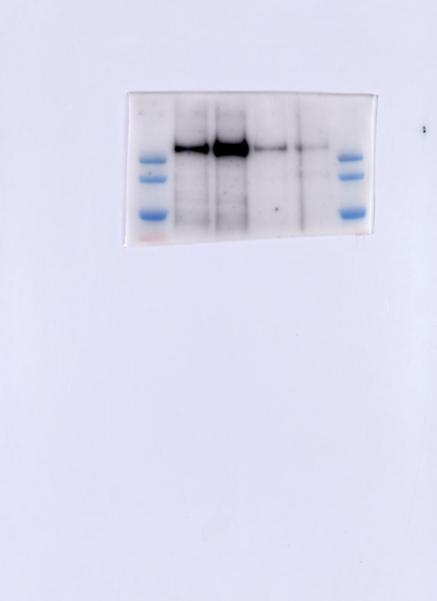


U937

SKM1

THP1

NOMO1

GAPDH MLL^C180^

**Fig. 4j** FLAG-MMP2 was expressed in THP1 cells and Co-IP was performed.


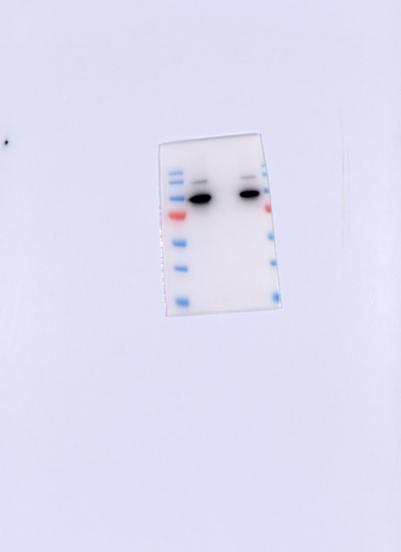

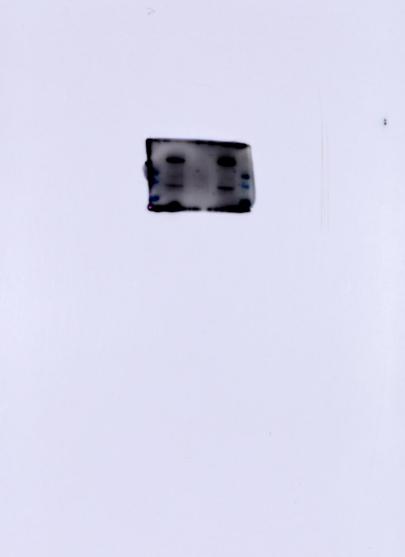

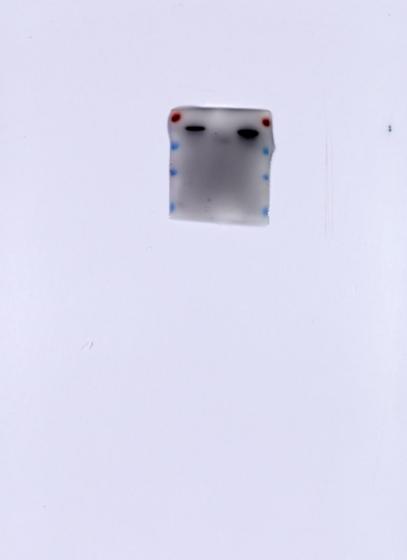


170kDa

130kDa

93kDa

input

igG

FLAG-IP

MMP2 eIF3η CCT2

**Fig. S4b** Immunoblots of MLL^C180^ in other MLL-r AML cell lines.

**
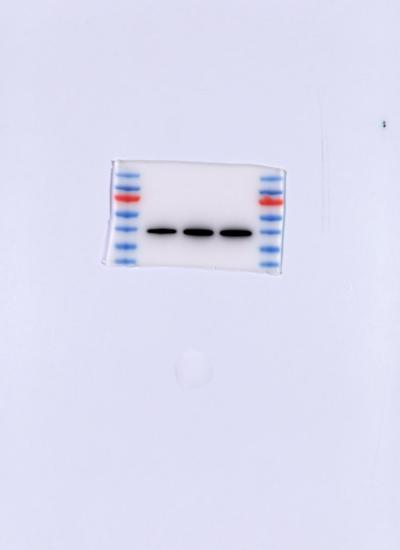

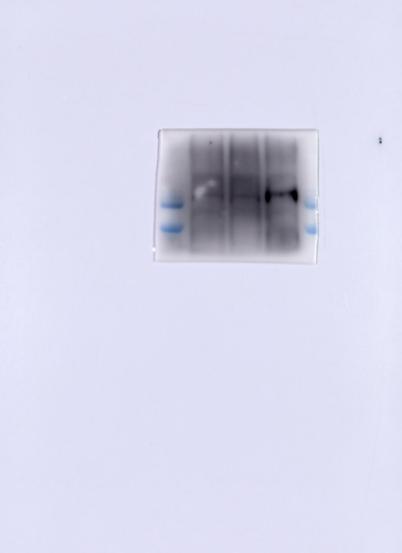
**

MOLM13

MV411

SHI1

GAPDH MLL^C180^

**Fig. S4d** MMP2 and H3K4me3 in SKM1 cells with treatment of MM-102.


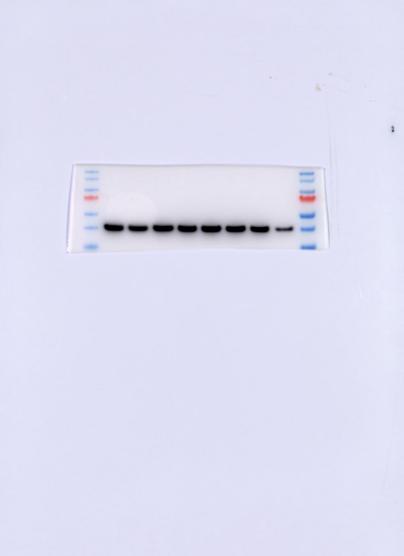

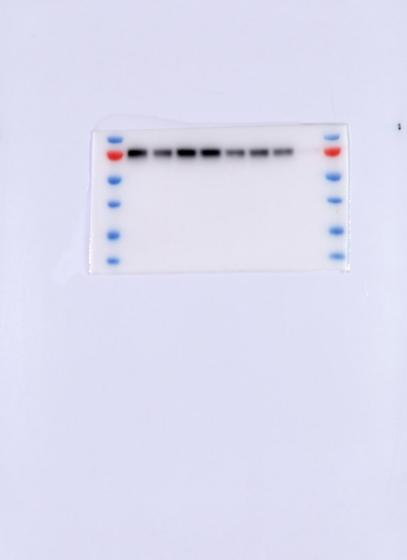

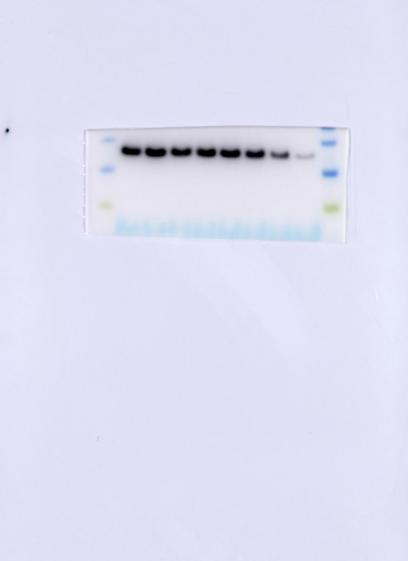


μM

MM-102 24h

100

50

20

10

5

DMSO

DMSO

100μM 12h

GAPDH MMP2 H3K4me3
